# Dual trigger *vs*. Conventional trigger outcomes in *In Vitro* Fertilization. Systematic review and meta-analysis

**DOI:** 10.5935/1518-0557.20220035

**Published:** 2023

**Authors:** Virginia González González, Alejandra Mayoral Triana, Irene Serrano García, Sara Osado Nieto, Marta Calvo Urrutia, Ignacio Cristóbal García, Teresa Gastañaga-Holguera

**Affiliations:** 1 Instituto de Salud de la Mujer. Hospital Clinico San Carlos, Madrid, Spain

**Keywords:** dual trigger, conventional trigger, *in vitro* fertilization, GnRH agonist, oocyte maturation

## Abstract

**Objective:**

The aim of this study is to analyze the efficacy of the dual trigger (human chorionic gonadotropin (hCG) + GnRH agonists) compared to the conventional trigger (hCG) in terms of oocyte retrieval (number and oocyte maturity), fertilization rate or number of embryos with two pronuclei, number of high-quality embryos, number of transferred embryos, number of cryopreserved embryos, implantation rate, positive β-hCG rate, ongoing pregnancy rate, abortion rate, and live birth rate.

**Methods:**

This search performed in this systematic review included all literature published in the PubMed database of studies on controlled ovarian stimulation with dual trigger compared with conventional trigger. The meta-analysis included clinical trials and prospective cohort studies.

**Results:**

Statistically significant differences between groups (dual trigger vs. hCG trigger) in terms of number of oocytes retrieved and live birth rate favored the dual trigger protocol. No statistically significant differences were found in the other studied variables. A tend favoring the dual trigger protocol was observed in all studied parameters.

**Conclusions:**

Dual trigger seems to be more effective in GnRH antagonist cycles in terms of embryo and pregnancy outcome.

## INTRODUCTION

As part of a standard controlled ovarian hyperstimulation (COH) regimen, final oocyte maturation and resumption of meiosis are generally triggered by a bolus injection of human chorionic gonadotropin (hCG) (5000-10,000 IU) administered as close to the theoretical time of ovulation as possible, which is usually 36 hours before oocyte retrieval (Orvieto, 2015; Ludwig *et al*., 2003).

Gonen *et al*. (1990) demonstrated that ovulation could also be triggered by the administration of a bolus injection of GnRH agonists (aGnRH) to cause the release and mimic the natural increase of endogenous LH and FSH, thus making it a more physiological method. Various recent studies have compared the activating effect of hCG *versus* aGnRH in in vitro fertilization (IVF) treatment cycles.

Recently, a new treatment mode has been implemented in routine clinical practice in patients with empty follicle syndrome (EFS) and low responders. The dual trigger consists of a combination of a single dose of GnRH agonist and a dose of hCG administered together (Kasum *et al*., 2016). A modification of the dual trigger, the so-called double trigger, consists of the co-administration of aGnRH and hCG for final oocyte maturation, 40 and 34 hours before ovum-pick up (OPU), respectively (Beck-Fruchter *et al*., 2012). These methods combine the advantage of both: (1) the prolongation of the time between the activation of ovulation and the puncture; and (2) the action of the GnRH agonist with the consequent simultaneous induction of an FSH peak (Haas *et al*., 2014).

This meta-analysis aimed to analyze the efficacy of the dual trigger compared to the conventional trigger protocol and to determine potential differences in the outcomes of the two trigger protocols.

## MATERIAL AND METHODS

### Search methods

Searches for articles in the PubMed database were conducted in February 2021 using the following keywords: double trigger or dual trigger. In order to focus the search to publications in the field of Assisted Reproduction, the following terms were added: “In Vitro Fertilization” and “IVF”. The search included only articles in English and Spanish. A specific publication time interval was not defined.

### Inclusion criteria

Quantitative analysis included randomized controlled trials (RCTs) and prospective cohort studies that compared the outcomes of dual versus hCG trigger in women undergoing IVF were included. Duplicate search results were excluded. Publications that clearly were not relevant based on the review titles and/or abstracts were excluded. Records that did not meet any of the inclusion criteria were excluded.

### Objectives

Various stimulation protocol outcomes were analyzed in order to determine the effects of dual versus conventional triggering. Primary outcomes included factors resulting directly from the techniques employed (number of oocytes retrieved; number of mature oocytes (MII) retrieved; number of two pronuclei (2PN) embryos obtained; and number of high-quality embryos). Other variables such as number of embryos transferred, implantation rate, Beta-HCG rate, ongoing pregnancy rate, abortion rate, and live birth rate were considered as secondary outcomes.

### Study variables

- Descriptive variables:

- Type of study
- Characteristics of the included patients (Age and BMI)
- Controlled ovarian hyperstimulation regimens and dosage
- Duration (days) of stimulation
- Estradiol (E2) levels on trigger day

Primary outcomes:

- Total number of oocytes retrieved per cycle
- Total number of metaphase II (MII) oocytes retrieved per cycle
- Total number of embryos with 2 pronuclei (2PN) obtained per cycle
- Fertilization rate
- Number of high-quality embryos on day 3.

Secondary outcomes:

- Number of embryos transferred
- Cycle cancellation rate
- Implantation rate
- Ongoing pregnancy rate
- Live birth rate
- Abortion rate

### Data extraction and quality assessment

Three reviewers independently extracted data from the included studies. The methodological quality of each study was assessed using the risk-of-bias assessment tool outlined in the Cochrane Handbook for Systematic Reviews of Interventions (Higgins & Green, 2011). Three reviewers subjectively reviewed all studies and assigned values of “low risk”, “high risk”, or “unclear” to the following: patient selection bias, intervention classification bias, bias due to deviations from intended interventions, bias due to missing data, bias in measurement of outcomes, reported result selection bias. Discrepancies between the results were resolved by consensus between the reviewers.

### Statistical study

A first statistical comparison of the outcome variables was made for ovulation induction with dual versus conventional trigger. For the analysis of dichotomous variables, Odds Ratios (OR) with 95% confidence intervals (95% CI) were used. CIs (confidence intervals) that included the null value (1) were considered not statistically significance, since there was insufficient evidence to reject the null hypothesis at the given confidence level. For the analysis of continuous variables, the Difference of Means (MD) with 95% confidence intervals (95% CI) was used. A mean difference greater or less than 0 represented a statistically significant difference. Pooled effects were calculated, and a two-sided *p* value<0.05 was considered to indicate statistical significance. The random effects model was used to combine the results according to their heterogeneity, applying the I^2^ statistical test. Heterogeneity determined based on the I^2^ statistic was defined as follows: 0-24%=no heterogeneity; 25-49%=moderate heterogeneity; 50-74%=large heterogeneity; and 75-100%=extreme heterogeneity. Publication bias analysis was not performed, since the number of studies was too little to allow the detection of an asymmetric funnel.

## RESULTS

### Study selection

**Figure 1:**
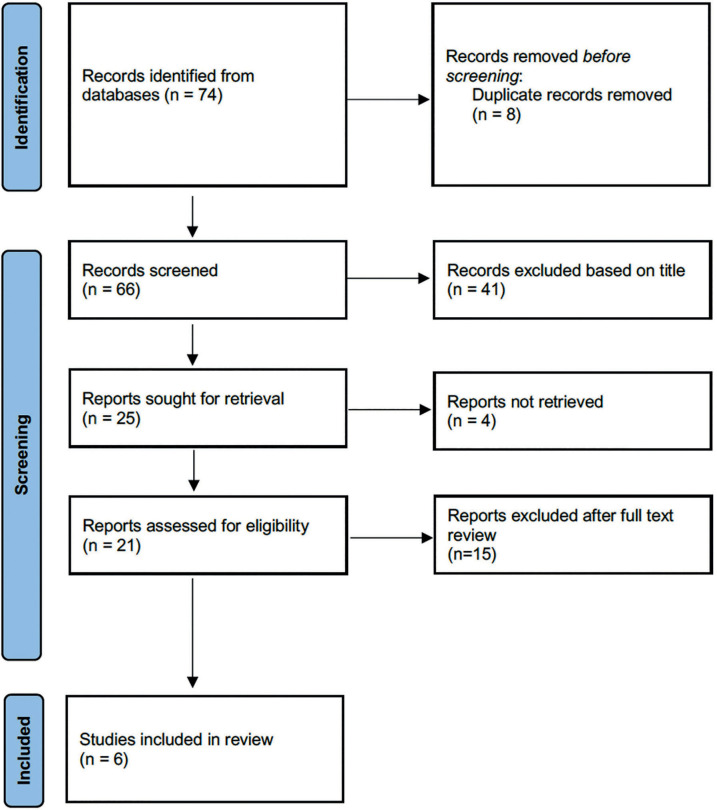
PRISMA flow diagram.

The article selection process is summarized in the PRISMA flow diagram in Figure 1. Initially, with the combination of keywords previously described, 74 articles were included. Eight were excluded for duplication. A total of 66 were selected, 41 of which were excluded during the analysis of titles. The full text of 21 of the 25 remaining articles was reviewed (four were not retrieved). Six articles were included (Schachter *et al*., 2008; Kim *et al*., 2014; Decleer *et al*., 2014; Mahajan *et al*., 2016; Maged *et al*., 2021; Haas *et al*., 2020) in quantitative or meta-analysis.

### Main characteristics of the studies

Table 1 shows the different articles included in the study, the type of study described in the articles, and the total number of participants in each group. Four of the six studies included were RCTs. The remaining two were observational prospective cohort studies. Table 2 shows the main characteristics of the patients in both groups of the different studies.

**Table 1:** Main characteristics of the studies.

Study	Type of study	N. total	N. dual group	N. hCG goup
Schachter *et al*., 2008	Randomized controlled trial	200	97	103
Kim *et al*., 2014	Observational. Prospective cohorts.	120	60	60
Decleer *et al*., 2014	Observational. Prospective cohorts.	120	61	59
Mahajan *et al*., 2016	Randomized controlled trial	76	38	38
Maged *et al*., 2021	Randomized controlled trial	160	80	80
Haas et al., 2020	Randomized controlled trial	155	85	70

N: Number of patients in

**Table 2:** Characteristics of the patients included.

Study	Age - Dual group (years)	Age - hCG group (years)	BMI - Dual group (kg/m^2^)	BMI - hCG group (kg/m^2^)
Schachter *et al*., 2008	33.7±5.6	34.7±4.7	N/A	N/A
Kim *et al*., 2014	36.2±3.7	35.8±3.8	21.7±2.0	21.4±2.2
Decleer *et al*., 2014	30±3.6	30.5±4.1	23.8±4.6	23.5±5.1
Mahajan *et al*., 2016	32±5	33±4	25.8±3.9	24.2±3.2
Maged *et al*., 2021	39.1±2.5	38.9±2.2	27.3±1.8	26.9±1.4
Haas *et al*., 2020	36±2.5	35.3±2.5	24±3.2	23.7±5.0

### Assessment of risk of bias in the included studies

**Figure 2:**
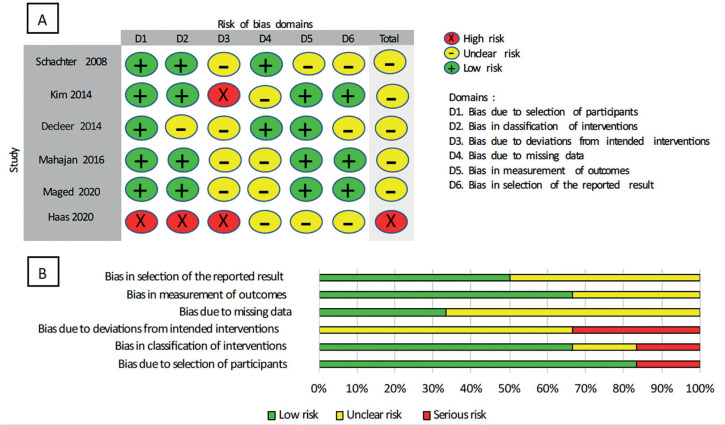
Risk-of-bias summary. A: Methodological quality items for each included study B: Methodological quality items presented as percentages across all included studies.

**Figure 3:**
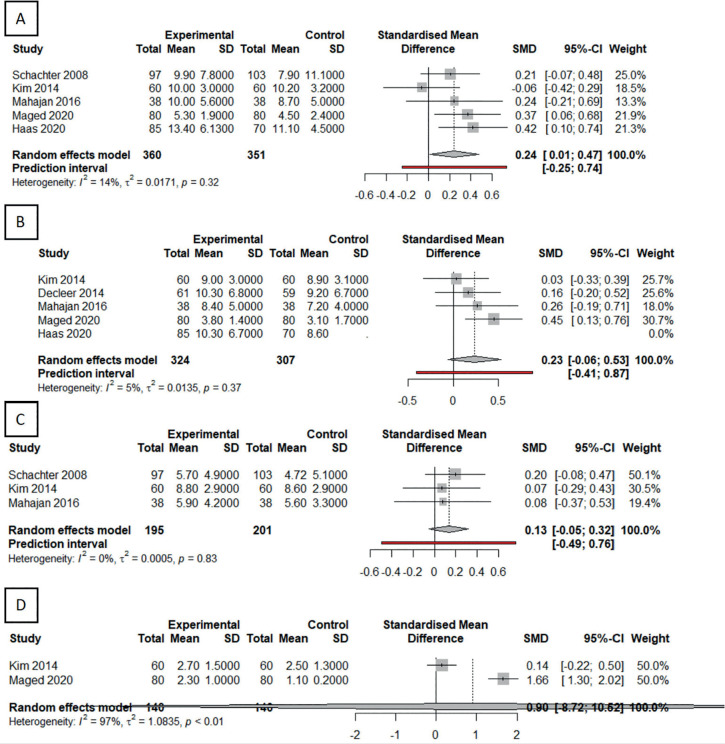
Forest plots comparing primary outcomes between dual triggering and hCG-alone triggering. A: Number of oocytes retrieved. B: Number of mature oocytes (MII) retrieved. C: Number of two pronuclei (2PN) embryos obtained. D: Number of high-quality embryos.

The details for assessing bias risk are presented in Figure 2 and Figure 3. All four RCTs were rated as having low risk of bias related to random sequence generation. Risk was rated as high in the observational studies due to patient selection practices.

### Controlled ovarian hyperstimulation regimens

All studies followed a GnRH antagonist protocol. Despite using the same type of COH protocol, the type of stimulation used by the different studies was very different, with different regimens and drugs, as shown in Table 3.

**Table 3:** Controlled ovarian hyperstimulation regimens.

Study	Type of protocol	Dual trigger group	hCG group
Schachter *et al*., 2008	Antagonist	hMG + Cetrorelix	HMG + Cetrorelix
Kim *et al*., 2014	Antagonist	150-225 UI rFSH + Cetrorelix	150-225 UI rFSH + Cetrorelix
Decleer *et al*., 2014	Antagonist	200 UI rFSH + Ganirelix	200 UI rFSH + Ganirelix
Mahajan *et al*., 2016	Antagonist	FSH + hMH-HP + Cetrorelix	FSH + hMH-HP + Cetrorelix
Maged *et al*., 2021	Antagonist	300 UI r-FSH + 150 UI LH+FSH + Cetrorelix	300 UI r-FSH + 150 UI LH+FSH + Cetrorelix
Haas *et al*., 2020	Antagonist	150-225 UI r-FSH + Ganirelix	150-225 UI r-FSH + Ganirelix

Table 4 shows the drugs and dosages used in the different studies for COH (total dose of FSH administered was cited in half of the studies) and the trigger protocol; all used hCG as the conventional trigger. Two studies used 250 µg / 6500 IU of hCG (equivalent total dose); two studies used a higher dose of 10,000 IU of hCG (Maged *et al*., 2021; Haas *et al*., 2020); and two studies (Schachter *et al*., 2008; Decleer *et al*., 2014) used a dosage of 5000 IU, which was lower than the dose used in the rest of the studies. Kim *et al*. (2014) was the only study in which placebo was added to the conventional hCG trigger.

**Table 4:** Interventions and dosage in COH and triggering.

Study	Dual group		hCG group	
	Total FSH dose	Triggering	Total FSH dose	Triggering
Schachter et al., 2008	N/A	0.2 mg triptorelin + 5000 UI hCG	N/A	5000 UI hCG
Kim et al., 2014	1879.4±457.2	0.1 mg triptorelin + 250 µg hCG	1859.6±462.8	250 µg hCG + placebo
Decleer et al., 2014	2083±590	0.2 mg triptorelin + 5000 IU hCG	2006±457	5000 UI hCG
Mahajan et al., 2016	2851.6±573	0.2 mg triptorelin + 250 µg hCG	2879.6±809.9	250 µg hCG
Maged et al., 2021	N/A	0.2 mg triptorelin + 10000 UI hCG	N/A	10000 UI hCG
Haas et al., 2020	N/A	1 mg leuprolide acetate + 1000UI hCG	N/A	1000UI hCG

N/A: Not applicable

In all studies IVF / ICSI techniques were used according to laboratory criteria, as shown in Table 5, except for Mahajan *et al*. (2016), in which ICSI was performed as the fertilization technique in all patients. In the studies in which reference was made to luteal phase support, progesterone supplementation was prescribed, although with disparities in presentation and dosage (Table 5). The study by Mahajan *et al*. (2016) did not mention luteal phase support

**Table 5:** Type of fertilization techniques used and luteal phase support treatment.

Study	Fertilization technique		Luteal phase support treatment	
	Dual trigger group	hCG group	Dual trigger group	hCG group
Schachter *et al*., 2008	IVF/ICSI	IVF /ICSI	400 mg/d progesterone	400 mg/d progesterone
Kim *et al*., 2014	IVF/ICSI	IVF/ICSI	90 mg progesterone vaginal gel (8%) / d	90 mg progesterone vaginal gel (8%) / d
Decleer *et al*., 2014	IVF/ICSI	IVF /ICSI	200 mg/8 h progesterone	200 mg/8 h progesterone
Mahajan *et al*., 2016	ICSI	ICSI	N/A	N/A
Maged *et al*., 2021	IVF/ICSI	IVF /ICSI	400 mg/12h progesterone	400 mg/12 h progesterone
Haas et al., 2020	IVF/ICSI	IVF /ICSI	400 mg/d progesterone	400 mg/d progesterone

N/A: Not applicable

IM: Intramuscular injection

Four of the studies specified the duration of stimulation (Table 6) and peak serum estradiol on the day of hCG administration.

**Table 6:** Days of stimulation and peak serum estradiol on hCG day.

Study	Days of COH		Peak serum estradiol (pmol/l)	
	Dual trigger group	hCG group	Dual trigger group	hCG group
Schachter *et al*., 2008	N/A	N/A	6054.04±3495.11	6461.56±4148.61
Kim *et al*., 2014	9.6±1.3	9.4±1.1	N/A	N/A
Decleer *et al*., 2014	11.7±2.1	11.4±1.7	N/A	N/A
Mahajan *et al*., 2016	10±1	10,3±1,4	7790.21±3617.37	6304.06±3861.14
Maged *et al*., 2021	13.4±1.2	13.1±1	4264.99±1769.59	3575.89±1545.63
Haas *et al*., 2020	N/A	N/A	8120 (6980-8920)	6818 (5610-7240)

N/A: Not applicable

### Primary outcomes

Statistically significant differences between dual and hCG trigger were observed in terms of total number of oocytes retrieved in four of the five studies evaluated. The dual trigger protocol yielded a greater number of retrieved oocytes in all cases minus Kim *et al*. (2014), (SMD=0.24, 95% CI=0.01, 0.74). The meta-analysis of the five studies found that total heterogeneity was not statistically significant (I2=14%, *p*=0.32), showing that the studies were homogeneous (Figure 3A).

Although an increase in the number of retrieved MII oocytes was observed in the dual trigger groups in all studies, no statistically significant differences were found (Figure 3B).

No statistically significant differences were observed between dual trigger and hCG in the number of two pronuclei (2PN) or fertilized embryos. Nevertheless, outcomes favoring the dual trigger protocol were observed in all studies (Figure 3C). No difference was seen in the number of high-quality embryos between the two groups (Figure 3D), although this variable was described in only two studies.

Only Maged *et al*. (2021) looked into fertilization rates, which were higher in the dual trigger (69.50%) compared with the conventional trigger (59.8%) group. Cancellation rates were lower in the dual trigger group (6/80; 7.5% *vs*. 16/80; 20%), although none of the other studies examined this variable.

Decleer *et al*. (2014) were the only to analyze the number of patients with the possibility of cryopreserving embryos (33/61 in the dual and 21/59 in the conventional trigger group). They also evaluated the number of cryopreserved embryos, with 2.2±2.9 in the dual group and 1.5±2.9 in the control group.

### Secondary outcomes

**Figure 4:**
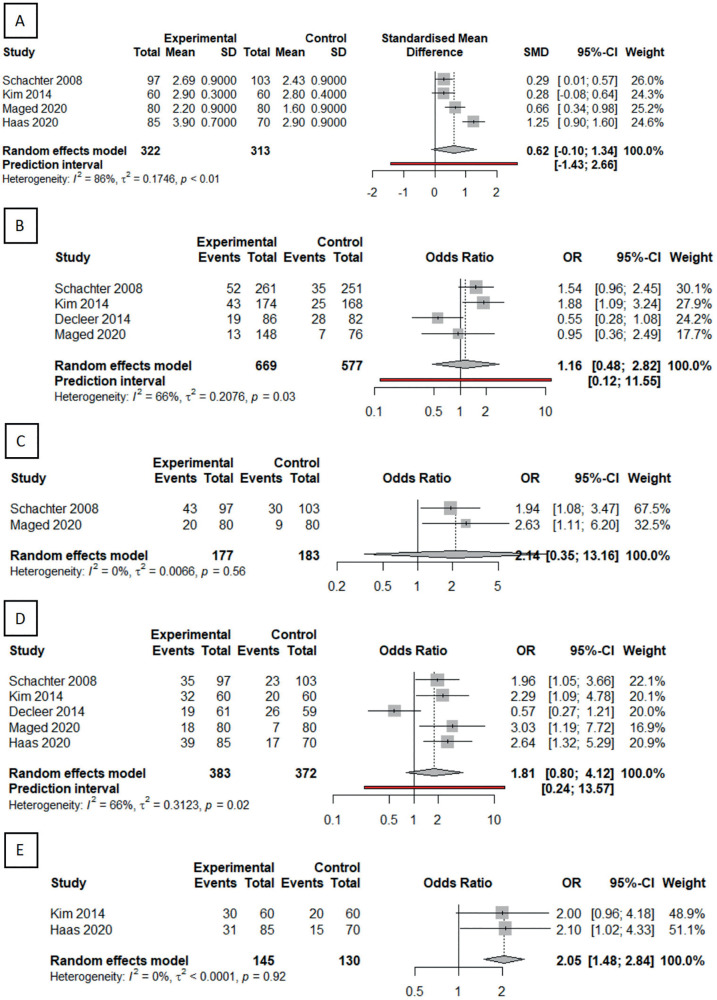
Forest plots comparing secondary outcomes between dual triggering and hCG-alone triggering. A: Number of embryos transferred. B: Implantation rate. C: Beta-HCG rate. D: ongoing pregnancy rate. E: New born rate.

No statistically significant differences were observed in the number of transferred embryos. Nevertheless, all studies described increased rates in the dual trigger group. In fact, Maged *et al*. (2021) and Haas *et al*. (2020) reported statistically significant differences in their analyses (Figure 4A). No differences were observed in implantation rate (Figure 4B) or β-HCG rate (Figure 4C). Four of the five studies described non-significant ongoing pregnancy rate increases (Figure 4D).

A significant live birth rate increase (Figure 4E) was see in the dual trigger group (OR=2.05, 95%CI=1.48, 2.84). Total heterogeneity was not statistically significant (I2=0%).

There was no reference to abortion rates in any of the studies.

## DISCUSSION

The meta-analysis of the data from the studies showed that the use of dual activation in women undergoing IVF presented a significant difference in favor of the dual trigger protocol when compared to hCG activation in terms of number of oocytes retrieved and live birth rate. No statistically significant differences were found for the other analyzed variables, although a trend favoring the dual trigger was identified in every parameter considered.

All studies reported an increase in the number of total oocytes retrieved and in the number of mature oocytes favoring the dual trigger. They also reported a higher number of high-quality embryos with the dual trigger protocol compared with the conventional trigger. Nevertheless, statistically significant differences were observed only in the total number of oocytes. Despite the increased number of mature oocytes and high quality embryos associated with the dual trigger, no statistically differences were observed in the analysis, with an MSD of 0.23 (IC: 0.06 - 0.53) in the study of mature oocytes (Figure 3B).

This might be explained by the ability of aGnRH to induce the release of both endogenous LH and follicle-stimulating hormone (FSH), which mimic more physiologically the increases seen in natural cycles.

One of the mechanisms that might explain the increase in pregnancy rate is the enhancement in receptivity and implantation due to the extra-pituitary effects of the GnRH molecule, which play a crucial role in endometrial receptivity, embryo implantation, and trophoblast invasion. The expression of HOXA-10, which has a role in modulating endometrial receptivity, is reduced in endometrial tissues in antagonist cycles (Oliveira *et al*., 2016).

Possible corpus luteum deficiency and luteal phase insufficiency, characterized by inadequate progesterone secretion, may induce early pregnancy loss when aGnRH is administered for oocyte maturation in GnRH antagonist cycles. The lack of a correlation between the intensity of luteal phase support and pregnancy outcome might suggest that, with dual activation, the hCG bolus injection plays a more important role in rescuing corpus luteum function than progesterone supplementation. This finding supports dual activation as an effective strategy to solve the luteal defect and restore endometrial receptivity (Oliveira *et al*., 2016; Chern *et al*., 2020).

A disadvantage of dual activation is the possible increased risk of ovarian hyperstimulation syndrome (OHSS) due to the use of hCG. However, the fact that this type of trigger occurs especially in women with low ovarian response means that the risk of OHSS is lower than that of the general population. Nevertheless, since this variable was not analyzed in the included studies, we cannot say whether higher OHSS rates were seen in the dual trigger group (Oliveira *et al*., 2016; Chern *et al*., 2020). Besides, existing evidence does not provide a clear association between dual trigger and risk of OHSS, particularly in patients at high risk of OHSS. Further studies and controlled clinical trials with careful patient selection are required to clarify the risk of OHSS after double or dual activation, and its potential relationship with activation with hCG or aGnRH alone, and whether risk is associated with dosage.

Although dual trigger appears to yield better results in IVF cycles, more controlled clinical trials should be performed to analyze the possible differences between dual and conventional trigger protocols with hCG.

## LIMITATIONS

The present meta-analysis has a series of limitations that deserve consideration. First, only six studies were eligible for inclusion and not all reported results for the analyzed variables. Second, the quality of the studies was only moderate, since blinding of participants and assessors was not performed or specified. Besides, the drugs used for the dual trigger differed between studies, although the regimes used were similar. Finally, the live birth rate, probably the most important outcome of IVF treatments, was only reported in two of the six meta-analyzed studies.

## CONCLUSION

In conclusion, the dual trigger or “dual activation”, described as a more physiological approach, seems to produce more favorable outcomes in COH cycles in terms of oocyte parameters and pregnancy outcomes. The dual trigger might increase the number of total and mature oocytes and high-quality embryos, and appears to improve pregnancy and live birth rates in IVF cycles with GnRH antagonists. However, it does not seem to affect the implantation rate or the positive Beta-HCG rate.

**Funding details:** This work was performed without financial support.
